# Correction: (In)Visible illness: A photovoice study of the lived experience of self-managing rheumatoid arthritis

**DOI:** 10.1371/journal.pone.0250451

**Published:** 2021-04-14

**Authors:** Susie Donnelly, Anthony G. Wilson, Hasheem Mannan, Claire Dix, Laura Whitehill, Thilo Kroll

## Notice of Republication

Incorrect versions of all Supporting Information files were published in error. This article was republished on April 6, 2021, to correct for this error. Please download this article again to view the correct version.
