# Supplementary material for: (In)Visible illness: A photovoice study of the lived experience of self-managing rheumatoid arthritis
Source: PLoS One. 2021 Mar 8;16(3):e0248151. doi: 10.1371/journal.pone.0248151 (PMC7939378; doi:10.1371/journal.pone.0248151)
Supplement: S1 Appendix — (DOCX) [file pone.0248151.s001.docx]

**S1 Appendix: Participant Information Leaflet and Consent Form**

**INFORMATION FOR PARTICIPANTS**

**Study Title:** Self-managing Rheumatoid Arthritis: A Photo Voice Study

*You are invited to participate in a research study.*

*Thank you for taking time to read this.*

This study uses photography to share people’s experience of living with rheumatoid arthritis (RA). You will be asked to take photos and discuss your daily life self-managing the condition. Here are some examples of the type of photos used in photo voice:


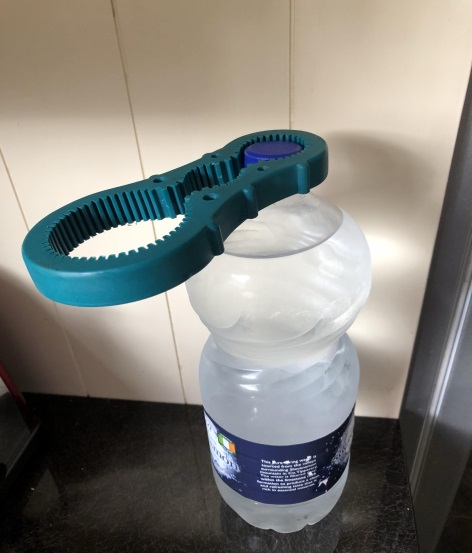


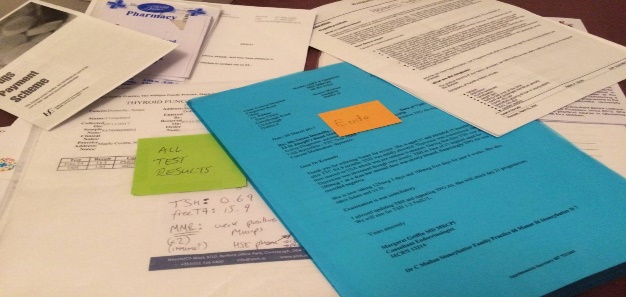


*“Oh my god! The paperwork. It’s hard to keep track of the prescriptions, referrals, lab results, insurance…Thankfully my partner helps with some of this.”*

*“This is a very simple device I use to open bottles as I don’t have the strength to open bottle tops.”*

Through your photos, you will share your experience with other participants and if you wish, take part in a photo exhibition. The findings of the study will be shared with relevant people that influence your care (such as researchers, health care practitioners or technical experts). As a group we will discuss how to develop solutions to better support people to self-manage RA in the future.

**Why have I been invited?**

You have been invited because you are living with rheumatoid arthritis and have not attended a self-management programme. No prior experience, skills or special equipment is necessary to take part.

**Who is organising the research study?**

The study is organised by Susie Donnelly PhD, sociologist with the Centre for Arthritis Research and the School of Health Systems, University College Dublin (UCD), and Professor Gerry Wilson, School of Medicine, UCD.

**What will happen to me if I take part?**

If you agree to take part, you will be asked to take part in three workshops, an interview and a photo exhibition over a period of about 6 months. Workshop locations will be readily accessible with good public transport links and disabled parking facilities.

*Introduction and Training Workshop* (Local Area / Region)*:* The study will be explained by the researcher. A photographer will give tips and advice. An easy to use camera will be provided and you will be shown how to use it. You do not need to return the camera after the study. If you prefer, you can use your own camera phone (but only if it produces photos of sufficient quality). You will be asked to spend one week taking photos. You may take as many photos as you like.

*Interviews* (Local Area / Region)*:* Afterwards, the researcher will contact you to arrange an interview to talk about your experiences. You will show the researcher photos that best reflect the theme of the study and that you are comfortable to share. This interview will be approx. 1-hour and audio recorded. It will take place at your home or a place of your convenience.

*Review of Photos Workshop* (Local Area / Region)**:** You will be invited to meet with fellow participants and share your photos. You will decide which photos, if any, you wish to show to others. As a group, you will work together to identify the main challenges and solutions that have emerged and you will agree upon what solution is most important to develop in order to support the self-management of RA. This event will be recorded to assist the researcher in note taking.

*Photo Exhibition* (Dublin). The purpose of this meeting will be to organise a photo exhibition. The group will decide how this should be done (for example, which images to display, who should be invited to the event, and where/when the exhibit should take place).

*Finding Solutions Workshop* (Dublin): The researcher and participants will share the findings of the study and discuss the solutions with relevant people that influence your care (such as researchers, health care practitioners or technical experts). This event will be audio recorded to assist the researcher in project outputs.

At the end of this study, the research team will seek future funding to develop the solution.

**What are the possible benefits of taking part?**

While there is no intended clinical benefit to taking part in this study, however you may enjoy the opportunity to meet other people with the same condition, share your experience, discuss solutions and learn more about photography.

**What are the possible risks of taking part?**

There are no obvious risks associated with taking part in this study. Some people may not enjoy reflecting on their struggles with a chronic illness. They can withdraw at any point.

**What if I want to withdraw from the study?**

You are completely free to opt out of the study at any point without giving reason. We just ask that you let a member of research team know by phone or email.

**Will my expenses be covered?**

At each workshop, you will be given a €25 multi-store gift card (One-for-All) to contribute towards the cost of travel expenses. You will also be given a camera (valued at approx. €50) which will be yours to keep after the study.

**Confidentiality – who will know I am taking part in the research study?**

No one will know you are taking part expect for the researchers and the other participants. Data from this study will be used in study outputs. Your identity will not be revealed at any point. All information collected is strictly confidential and will be stored safely on encrypted computers or secure servers as per university policy. Upon entering the study, you will be assigned an ID number and only this ID number will appear beside your information. Your name will not appear beside any information we collect about you. Only a member of the research team will have access to the key that links participants’ names to their ID numbers. This key will be destroyed once the study is completed.

You own the right to any data (e.g. photos or similar material) that you create during the study. Data protection guidelines state that you have the right to request to see any data that has been collected relating to you. At the end of the study or if you decide to withdraw from the study you can request to see this data by contacting the researcher.

Additionally, you always have the right to request that all data stored relating to you be destroyed. You can do this at the end of the study or if you withdraw at any point in the study. However, you cannot continue in the study if you request data be destroyed.

You can find information about your rights under data protection law here:

https://www.dataprotection.ie/docs/A-guide-to-your-rights-Plain-English-Version/r/858.htm

Alternatively, contact the Data Protection Commission (email: info@dataprotection.ie / Lo call: 1890 252 231).

**Who has approved the study?**

The Ethics Committee at University College Dublin has approved this study.

**Who is funding this research?**

Funding was awarded from the UCD Wellcome Trust Institutional Strategic Support Fund which was financed jointly by University College Dublin and the SFI-HRB-Wellcome Trust Biomedical Research Partnership as part of a Medical Humanities and Social Science Collaboration Scheme.

**What will happen to the results of the research study?**

Outputs from the research will be shared with participants (e.g. academic publications, presentations and reports). At any stage, the researcher would be happy to talk with you about either your own specific participation or the findings from the general study.

**What happens next?**

Please take time to review this information and please contact the researcher if you

have questions or wish to discuss further:


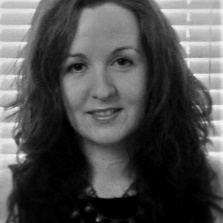
Susie Donnelly

School of Nursing, Midwifery and Health Systems

Room B113, UCD Health Sciences Centre

University College Dublin (UCD)

Belfield, Dublin 4

Email [removed] Tel: [removed] (direct line)


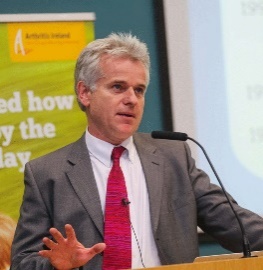


Professor Gerry Wilson

School of Medicine
University College Dublin (UCD)

Conway Institute
Belfield, Dublin 4

Email [removed] Tel: [removed]

*Thank you for taking the time to consider participating in this study and I look forward to speaking with you.*

**CONSENT FORM**

**Title of Research Study:** Self-Managing Rheumatoid Arthritis: A Photo Voice Study

**Name of Sponsor:** University College Dublin

*Please read carefully and* ***tick EACH box*** *to indicate agreement:*

| I confirm that I have read and understood the information leaflet for the above research study and received an explanation of the nature, purpose, duration, and foreseeable effects and risks of the research study and what my involvement will be |  |
| --- | --- |
| I have had time to consider whether to take part in this research study |  |
| My questions have been answered satisfactorily and I have received a copy of the Participant Information document |  |
| I understand that my participation is voluntary (my choice) and that I am free to withdraw at any time without my medical care or legal rights being affected |  |
| I agree that the data collected for the study will be used for the purpose set forth above, and processing by the study sponsor in an anonymous form to protect the confidentiality of my data. This will not waive any rights that I have under local law |  |
| I agree that my interview and a selection of my photos with captions will be archived with a digital repository subject to my name and all identifiable details removed |  |
| I understand that I will be asked to complete a separate consent form  in the follow-up interview about how my photographs will be used |  |
| I consent to being contacted by phone for the duration of the study |  |
| I understand I will be given a copy of this consent form, after signing it and a copy of the patient information leaflet |  |
| I agree to take part in the above research study |  |

……………………………… ……………………… ………

Name of Participant (in block letters) Signature Date

……………………………… ……………………… ………

Researcher Signature Date

*1 copy for participant, 1 copy for researcher*
