# Supplementary material for: (In)Visible illness: A photovoice study of the lived experience of self-managing rheumatoid arthritis
Source: PLoS One. 2021 Mar 8;16(3):e0248151. doi: 10.1371/journal.pone.0248151 (PMC7939378; doi:10.1371/journal.pone.0248151)
Supplement: S2 Appendix — (DOCX) [file pone.0248151.s002.docx]

## S2 Appendix: Overview of Group Workshop 1

In this three-hour workshop, participants will be introduced to one another and the photo assignment. They will be provided with basic training to use a camera, exercises in visual literacy, and instructions for taking photos.

**12:30- 1pm** Introduction (20-30 mins) [SD] & [CD]

**Objectives:** (i) Create a comfortable and friendly environment; (ii) Assess group interest and expertise in photography; (iii) Provide participants with a brief reminder of the study and methodology.

Introductions to one another and to the study.

1. Ice breaker exercises: i) Tell an interesting fact / story about yourself;
2. Brief intro to photovoice and the study – take questions

**1pm – 1:30pm** Visual literacy (30 mins) [CD]

**Objectives:** (i) Provide participants with basic visual literacy. Encourage them to think about interpreting meaning from images.

1. Talk about participant’s own experiences or lack of with cameras and taking pictures.
2. Ask what do images do best? Why are they powerful? (Moving us emotionally, communicating a message; giving a window into a world / a state of mind; telling a story/asking a question/piquing our interest).
3. Provide a selection of random images (e.g. from *National Geographic*, *Time* magazine) and arrange on the table. Invite participants to choose an image and then talk to the group about why they feel drawn to this image.
4. Divide into groups and ask participants to discuss their own personal experiences of RA and think about how they could capture it in an image.

*LUNCH / REFRESHMENT BREAK*

**2pm – 2:20pm** Photo Assignment (20 mins) [SD]

**Objectives:** (i) Instruct participants on the photo assignment (ii) Discuss ethical considerations

1. What am I being asked to do? And how should I do it?
2. Ethics and consent
   - Brief participants on issues, such as:
     - Consent; How / when / why to use consent forms for photographic subjects; what makes a subject identifiable?
     - Ownership / copyright of images and how images will be used
3. Take questions

**2:20pm – 3:20pm** Taking Photos (30-40 mins) [CD]

**Objectives:** (i) Ensure each participant has a camera and is comfortable using it; (ii) provide basic instruction in taking photos; (ii) provide overview of common technical and artistic considerations (e.g. framing, composition, being in focus, using the light).

1. Provide attendees with a camera and instructions (or check smartphones for appropriateness). [CD] & [SD] spend a few moments with each participant to ensure they can use the camera.
2. Photo taking exercise: Take portraits of each other / object in room. Simple exercise to get used to functions of the cameras.
3. Discuss technical and artistic considerations:
   1. First think about what it is you want to express. How to take an image that conveys something personal and does the above for you.
   2. What makes an image visually pleasing?
   3. Technically how to make your image work: (i) Being in focus (something to practice with the camera); (ii) In low light with certain phones it can be tricky for the camera to take a sharp image so play around and see how your camera copes; (iii) Lighting: Someone looking away from the light will have their face in deep shadow.
4. Second photo-taking exercise where they consider the above and take photo inside / outside venue.
5. Review photos with group (only if participant is comfortable / volunteers to share their photo).

**3:20-3:30pm** Closing (10 mins) **[SD]**

Before leaving, all participants should:

- Ask any final questions / raise any outstanding issues
- Have a folder pack with information and instructions on the assignment and how to use the camera
- Have a camera. If using a personal smartphone device, have it approved by team.
- Be comfortable with the device they are using and know what they need to do
- Complete an “Interview Availability” form to schedule interview with **[SD]**
- Have received a gift voucher
