# Supplementary material for: (In)Visible illness: A photovoice study of the lived experience of self-managing rheumatoid arthritis
Source: PLoS One. 2021 Mar 8;16(3):e0248151. doi: 10.1371/journal.pone.0248151 (PMC7939378; doi:10.1371/journal.pone.0248151)
Supplement: S3 Appendix — (DOCX) [file pone.0248151.s003.docx]

## S3 Appendix: Overview of Group Workshop 2

In this three-hour workshop, participants will work together to select photographs and share them with the rest of the group. They will also contextualize and codify their photographs, and identify emergent themes.

**Arrival and Debriefing** (15-20 min) [SD] & [CD]

**Objectives:**

- To give participants a chance to share their Photovoice experience and any challenges
- To have participants to go through their photographs and select the photographs they would like to share with the rest of the group.

1. Welcome back participants and hand them their nametags. Ask participants:
   1. Do I have your consent to audio record today’s session? (verbal, reconsent)
   2. How was your experience taking photographs? Did you have any concerns or difficulties?
2. Briefly share and discuss participants' experiences.
3. Give each participant an envelope with their printed photographs. If they have additional phots with them on the day, a printer is on hand.
4. Restate the research prompt: “the challenges and solutions to living with rheumatoid arthritis” and ask each participant to review their photos, and select any they would like to discuss with the group today.

**Establishing Ground Rules** (10 min) [SD]

Establish ground rules for the session to ensure respect for participants’ comfort and privacy. Note the ground rules on a flipchart and ask participants to be mindful of these rules, both during and outside the group discussion.

**Group Review and Analysis of Chosen Photographs** (30 mins) [SD]&[CD]

1. Divide participants into groups of two to three.
2. After everyone has selected their photographs, go around the circle and ask each

participant to present their photograph, one at a time, contextualizing the photograph by explaining why it was taken in the context of the research questions.

1. Ask them to think about the following questions in presenting their pictures and stories (e.g. SHOWED method).
2. Invite other members of the group to give their own perspective on the photograph after the participant has shared his/her initial story. Probe using questions such as: *What is your reaction to the story and photograph that was just presented? How are your experiences similar? How are your experiences different?*
3. Once all the participants have shared their photographs, summarize the main findings of the discussion session and some of the commonalities that arose.

**Caption development and approval** (20 mins) [SD] & [CD]

Encourage participants to write out a story/caption to accompany their photographs. Provide opportunity to develop captions in their own time and send to researcher at a later stage, if desired. Each caption, once developed, will be shared with the participants for approval prior to the exhibition.

*LUNCH / REFRESHMENT BREAK*

**Thematic analysis, Codifying and Voting** (1hr) [SD] & [CD]

The objective of this session is to identify and define emerging themes within the photographs.

1. Put the stories and photographs together and display them around the room in a mini-exhibition.

2. Give the participants a set of stickers and ask them to go around and put a sticker on at least one photograph that they like or feel best represents their perspective.

3. After the photographs have been shared and discussed, ask the group to categorize the photographs into major themes that they see to be emerging from the data, through discussion and physical manipulation of the photos. Probe using questions such as*: Do you see any similarities in the kind of topic/issue these photographs represent? Do some of these photographs address the same theme? Which ones? What kinds of themes are emerging?*

4. Begin to note these themes down on large sheets and place them on the floor so everyone

can see them. Start sorting the photographs according to the themes identified, as directed by

the participants. Ask participants to help you fit each photograph into its relevant theme.

5. Throughout the process of thematically arranging the photographs, ensure that other group members agree when somebody has arranged or rearranged photographs. If they disagree, ask for reasons and try to facilitate the process to reach a general agreement.

6. Summarize all the themes identified and defined during the session.

7. Note down the thematic area for each photograph on the photograph or in your notebook.

8. Ask them to sign an *Exhibition Release Form* to consent to the use of their photos and captions in a public exhibition.

**Exhibition Planning (25 mins)** [SD] & [CD] Dissemination strategies for the outputs from the project should be developed in partnership

with the participants. Present overview to group:

- Who to invite? Members of the community, policy-makers, the media, and other stakeholders.
- Where? Present short list of possible venues
- When? Provide short list of dates (this may be shaped by availability of venue)
- Requirements: Accessibility, well-serviced by public transport, neutral space.

**Closing (5 mins)** [SD] & [CD]

- Ask approx. 1 participant (from each group) to volunteer to be on an Exhibition Committee that will guide the exhibition process. These participants will work more closely with [SD] & [CD] to finalise details of the exhibition.
- Ask any final questions / raise any outstanding issues
- Distribute gift vouchers
- Thank participants and inform them of next steps.

*Source:* Adapted from, Mohammed, S., & Sajun, S. (2014) Photovoice Manual. DOI 10.13140/RG.2.2.17685.12006
