# Supplementary material for: (In)Visible illness: A photovoice study of the lived experience of self-managing rheumatoid arthritis
Source: PLoS One. 2021 Mar 8;16(3):e0248151. doi: 10.1371/journal.pone.0248151 (PMC7939378; doi:10.1371/journal.pone.0248151)
Supplement: S4 Appendix — (DOCX) [file pone.0248151.s004.docx]

## S4 Appendix: Phases of Thematic Analysis

**
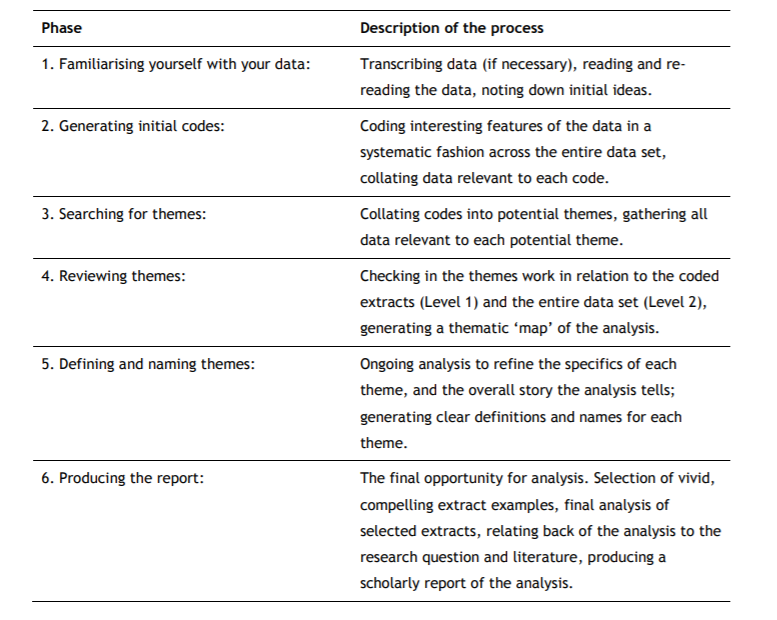
**

*Source:* Braun, V. and Clarke, V. (2006) Using thematic analysis in psychology. Qualitative Research in Psychology, 3 (2). pp. 77-101. ISSN 1478-0887
