# Supplementary material for: (In)Visible illness: A photovoice study of the lived experience of self-managing rheumatoid arthritis
Source: PLoS One. 2021 Mar 8;16(3):e0248151. doi: 10.1371/journal.pone.0248151 (PMC7939378; doi:10.1371/journal.pone.0248151)
Supplement: S5 Appendix — (DOCX) [file pone.0248151.s005.docx]

## S5 Appendix: Consolidated Criteria for Reporting Qualitative Research (COREQ)

| **Supplementary Table 1: COREQ checklist** | | |
| --- | --- | --- |
| **Domain 1: Research team and reflexivity** | | |
| **Personal Characteristics** | **Description** | **Location in manuscript (Section, page)** |
| 1. Interviewer/facilitator. Which author/s conducted the interview or focus group? | Interviews [SD], Workshops [CD] & [SD] | METHOD / Procedure, p. 7-9 |
| 2. Credentials. What were the researcher’s credentials? E.g. PhD, MD | Credentials of lead researcher [SD] as follows:  BA Journalism and Media Communications, HDip Sociology and Social Research, MSocSc Sociology, PhD Sociology. Prior postdoctoral position in Connected Health ethnography. | METHOD, p. 5-6 |
| 3. Occupation. What was their occupation at the time of the study? | Postdoctoral Research Fellow | METHOD, p. 5 |
| 4. Gender. Was the researcher male or female? | Female | - |
| 5. Experience and training. What experience or training did the researcher have? | [SD] has experience with photovoice methodology and qualitative research | METHOD, p. 5 |
| **Relationship with participants** | | |
| 6. Relationship established. Was a relationship established prior to study commencement? | No | METHOD, Inclusion Criteria and Recruitment, p. 6 |
| 7. Participant knowledge of the interviewer. What did the participants know about the researcher? e.g. personal goals, reasons for doing the research | Participants were briefed on the purpose of the study and understood that it was a research project conducted by [SD] as part of her postdoctoral fellowship. Participants reviewed the participant information  documentation prior to giving their written informed consent to be involved. | - |
| 8. Interviewer characteristics. What characteristics were reported about the interviewer/facilitator? e.g. Bias, assumptions, reasons and interests in the research topic | Researcher [SD] explained a key motivation to explore invisible illness was that she has a chronic autoimmune disease. Participants were informed that as well as public impact, academic publications were an important driver. | - |
| **Domain 2: study design** | | |
| **Theoretical framework** |  |  |
| 9. Methodological orientation and Theory. What methodological orientation was stated to underpin the study? e.g. grounded theory, discourse analysis, ethnography, phenomenology, content analysis | Photovoice / Participatory action research | ABSTRACT, p. 1 |
| **Participant selection** | | |
| 10. Sampling. How were participants selected? e.g. purposive, convenience, consecutive, snowball | Purposive | METHOD, Inclusion Criteria and Recruitment, p. 6 |
| 11. Method of approach. How were participants approached? e.g. face-to-face, telephone, mail, email | Four recruited were approached by a rheumatology health care professional in a hospital setting (i.e. rheumatology clinic).  Seven approached the researcher directly [SD] following public calls for study volunteers across a variety of public platforms (e.g. regional newspaper adverts, flyers, posting on social media groups and newsletters). | METHOD, Inclusion Criteria and Recruitment, p. 6 |
| 12. Sample size. How many participants were in the study? | 11 | ABSTRACT, p. 1 |
| 13. Non-participation. How many people refused to participate or dropped out? Reasons? | *Partial-participation:*  1 participant attended the first workshop and completed the semi-structured interview, but did not wish to participate in further group workshops. Upon discussion with the researcher, it was agreed that she would not be included in the exhibition but was happy for her interview data to be analyzed as part of the study and was expressly invited to attend the exhibition and participate in member-checking by post. She is therefore included in the reported sample size (n=11).  *Non-participation:*  1 participant attended the first workshop but thereafter dropped out due to family illness.  5 participants were enrolled at the hospital clinic (n=4) or over the phone with the researcher (n=1) but did not attend the first workshop.  A further two participants had intended to take part but were forced to drop out as they were unable to travel to the workshop location once it was announced. | - |
| **Setting** | | |
| 14. Setting of data collection Where was the data collected? e.g. home, clinic, workplace | Workshops were held in a privately hired meeting room at a hotel in a central accessible location in Dublin city centre.  Interviews took place at the participants home (n=7); a private meeting room (n=3), or semi-private meeting area (n=1). | METHOD / Procedure, p. 7-9 |
| 15. Presence of non-participants. Was anyone else present besides the participants and researchers? | [SD] & [CD] were present for all workshops.  [SD] conducted interviews alone. | - |
| 16. Description of sample. What are the important characteristics of the sample? e.g. demographic data, date | - | RESULTS, Table 1, p. 13 |
| **Data collection** | | |
| 17. Interview guide. Were questions, prompts, guides provided by the authors? Was it pilot tested? | The authors **[**SD], [TK], [HM] agreed upon a set of questions for the semi-structured interviews in advance. Following the first interview, adjustments were made to the order of questions. | - |
| 18. Repeat interviews. Were repeat interviews carried out? If yes, how many? | No | - |
| 19. Audio/visual recording. Did the research use audio or visual recording to collect the data? | Audio recording | - |
| 20. Field notes. Were field notes made during and/or after the interview or focus group? | Yes | - |
| 21. Duration. What was the duration of the interviews or focus group? | Workshops lasted 3 hours.  The average duration of interviews was 1 hour and 13 minutes (ranging from 54 minutes to 1 hour, 46 minutes). | METHOD / Procedure, p. 7-9 |
| 22. Data saturation. Was data saturation discussed? | No | - |
| 23. Transcripts returned. Were transcripts returned to participants for comment and/or correction? | Participants were contacted by email / post and invited to review a copy of their transcript. No participant requested their transcript. | - |
| **Domain 3: analysis and findings** | | |
| **Data analysis** |  |  |
| 24. Number of data coders. How many data coders coded the data? | Study participants coded visual data in Workshop 2 in collaboration with [SD] & [CD]. Further data coding was performed by [SD] in consultation with [HM, LW & TK] | Appendix B, p. 45-46  METHOD / Data Analysis, p. 9-10 |
| 25. Description of the coding tree. Did authors provide a description of the coding tree? | Yes | RESULTS, Figure 1. Thematic Map, p. 14 |
| 26. Derivation of themes. Were themes identified in advance or derived from the data? | Main themes were identified by participants in advance. | METHOD / Data Analysis, p. 9-10 |
| 27. Software What software, if applicable, was used to manage the data? | NVivo 12 | METHOD / Data Analysis, p. 9-10 |
| 28. Participant checking. Did participants provide feedback on the findings? | Yes, participants were invited to provide feedback on a summary of the analysis and thematic map (Figure 1). Four participants contacted the researcher [SD] and confirmed that the analysis was valid. Similarly, participants were invited to review a draft of this manuscript and three responded. Again, feedback was limited to confirming they were happy with the manuscript. | METHOD, Flowchart 1 p. 7  DISCUSSION, Strengths and Limitations, p.35-36 |
| **Reporting** | | |
| 29. Quotations presented. Were participant quotations presented to illustrate the themes / findings? Was each quotation identified? e.g. participant number | Quotations were presented to illustrate the themes. Each quotation was not identified. | - |
| 30. Data and findings consistent. Was there consistency between the data presented and the findings? | Yes | - |
| 31. Clarity of major themes. Were major themes clearly presented in the findings? | Yes | - |
| 32. Clarity of minor themes. Is there a description of diverse cases or discussion of minor themes? | Yes, subthemes are described in detail throughout the Findings section. | RESULTS, Visual and Narrative themes p. 13-30 |
